# *ATAD2* Upregulation Promotes Tumor Growth and Angiogenesis in Endometrial Cancer and Is Associated with Its Immune Infiltration

**DOI:** 10.1155/2022/2334338

**Published:** 2022-11-28

**Authors:** Can Wang, Yue Yin, Zhenxing Sun, Yiru Wang, Fei Li, Yan Wang, Zexue Zhang, Xiuwei Chen

**Affiliations:** Department of Gynecologic Oncology, Third Affiliated Hospital of Harbin Medical University, Harbin Heilongjiang Province 150000, China

## Abstract

**Background:**

Endometrial cancer is one of the three major gynecologic malignancies, and its incidence continues to rise. ATPase family AAA structural domain-containing protein 2 (ATAD2) is an ATPase protein, which is an independent factor for poor prognosis in endometrial cancer. However, its role in the disease is yet to be determined.

**Methods:**

The Tumor IMmune Estimation Resource (TIMER) database was used to assess ATAD2 expression in pan-cancer, and the relevance of ATAD2 expression in Uterine Corpus Endometrial Carcinoma (UCEC) in clinical settings was obtained using Gene Expression Profiling Interactive Analysis (GEPIA) and UALCAN analysis. In addition, the Human Protein Atlas database was used to assess ATAD2 protein expression in UCEC. Furthermore, *in vitro* molecular biology and *in vivo* functional experiments were employed to ascertain the effect of ATAD2 expression on tumor angiogenesis and tumor growth. UALCAN was used to screen for *ATAD2* coexpressed genes, and Sangerbox was utilized to perform Gene Ontology (GO) and Kyoto Encyclopedia of Genes and Genomes (KEGG) enrichment analyses of these coexpressed genes. Finally, the TIMER, Tumor Immune System Interaction and Drug Bank (TISIDB), and GEPIA databases were used to analyze the relationship between *ATAD2* and immune infiltration.

**Results:**

*ATAD2* is highly expressed in a variety of tumors, and in UCEC, it plays the role of a protooncogene. Basic experiments revealed that ATAD2 promotes vascular endothelial growth factor expression in endometrial cancer and affects tumor growth and angiogenesis. In addition, GO and KEGG enrichment analyses showed that *ATAD2*-associated genes were chiefly enriched in certain signaling pathways, such as herpes simplex virus 1 infection and that *ATAD2* was associated with immune infiltration in UCEC.

**Conclusion:**

Our findings suggest that *ATAD2* promotes tumor growth and angiogenesis in endometrial cancer. Furthermore, *ATAD2* is associated with immune infiltration and is a potential diagnostic and therapeutic target.

## 1. Introduction

Endometrial cancer is the sixth most common cancer that threatens women's health and ranks second in gynecologic tumors, next to cervical cancer. In 2020, there were 417,000 new cases and 97,000 deaths [1]. Approximately 10%–15% of patients with endometrial cancer are already in advanced stages at the time of diagnosis and, hence, have a very poor prognosis [2]. Although patients in the early stages of the disease can be treated with surgery and adjuvant therapy, there is no effective treatment for advanced disease [3]. Therefore, it is particularly important to explore new treatment methods and early diagnostic indicators.

ATAD2 (ATPase family AAA structural domain-containing protein 2) is a cancer testicular protein involved in multiple signaling pathways [4]. ATAD2 plays a role in various processes of cell survival, such as DNA replication [5], transcription, and cell cycle control activities [6]. Moreover, several studies have established that ATAD2 is overexpressed in many cancer types, including lung adenocarcinoma [7], breast cancer [8], colorectal cancer [9], gastric cancer [10], hepatocellular tumor [11], ovarian cancer [12], and cervical cancer [13]. Moreover, two structural domains in ATAD2, namely, the bromodomain and the ATPase structural domain, are “druggable” targets [14]. The research on small molecule inhibitors of ATAD2 has received extensive attention and has value for further research [15]. These investigations provide a basis for *ATAD2* as a new therapeutic and diagnostic target. In addition, our team found that high stage of Federation of Gynecology and Obstetrics (FIGO), poor pathological grading, extensive lymph node infiltration, deep myometrial infiltration, and high recurrence rate of endometrial cancer is associated with the high expression of ATAD2 [16]. In 2015, a study observed that the expressions of *E2F transcription factor 1* (*E2F1*), *E2F transcription factor 2* (*E2F2*), and *MYB proto-oncogene like 2* (*MYBL2*) are closely associated with *ATAD2*, which may be related to the invasion and progression of endometrial cancer [17]. These findings suggest that *ATAD2* is involved in and contributes to the development of endometrial cancer. However, the mechanism of its influence on the development of endometrial carcinogenesis has not been elucidated.

Tumor growth and metastasis depend on angiogenesis, which plays a key role in the process of tumor development, progression, and regression. More than a century ago, some German pathologists identified extensive neovascularization in the tumor, which signifies that the process has a pathogenic effect on cancer [18]. *VEGF* (*vascular endothelial growth factor*) has gradually surfaced over a century, and research has shown that it not only affects physiological angiogenesis but also influences pathological angiogenesis [19]. In brief, *VEGF*, a factor that could accelerate the proliferation of HUVEC cells, is overexpressed in many tumor types and promotes their occurrence and development [20, 21]. Although VEGF is highly expressed in many types of tumors, it may be the result of a variety of genetic and epigenetic modalities that induce it [22]. Owing to the extensive research on VEGF, the *VEGFA* (*vascular endothelial growth factor A*) inhibitor bevacizumab was authorized by the Food and Drug Administration for the first-line treatment of metastatic colorectal cancer in 2004 [23, 24]. Subsequently, the addition of bevacizumab to chemotherapy regimens for cervical cancer [25], ovarian cancer [26], endometrial cancer [27, 28], and several other tumors was also approved [29]. The use of VEGF inhibitors significantly improved the prognosis of patients [29]. In 2007, a team from the University of Texas found that high expression of VEGF in endometrial carcinoma was an independent poor prognostic factor. Later, an in situ mouse model of endometrial cancer was constructed and treated with the VEGF inhibitor bevacizumab alone and in combination with doxorubicin. The findings demonstrated that the long-term use of bevacizumab, especially in combination with doxorubicin, was highly effective in inhibiting neoplasm development and metastasis [30]. These results allude that *VEGF* is integral in the progression of endometrial neoplasm.

In this study, Tumor IMmune Estimation Resource (TIMER) was used for ATAD2-associated pan-cancer analysis, which was followed by the analysis of ATAD2 expression and clinical relevance in Uterine Corpus Endometrial Carcinoma (UCEC) using various biochemical tools. The effects of ATAD2 on UCEC tumor growth and angiogenesis were verified with *in vivo* and *in vitro* correlation experiments. Finally, UALCAN was used to screen *ATAD2* coexpressed genes, followed by Gene Ontology (GO) and Kyoto Encyclopedia of Genes and Genomes (KEGG) enrichment analyses using Sangerbox. Moreover, Immune System Interaction and Drug Bank (TISIDB), TIMER, and Gene Expression Profiling Interactive Analysis (GEPIA) were used to assess the correlation between ATAD2 and immune infiltration. This study demonstrated the positive effect of *ATAD2* on tumor growth in endometrial cancer and established its use as an early diagnostic marker and therapeutic target.

## 2. Materials and Methods

### 2.1. Bioinformatics Analysis

ATAD2 expression analysis in pan-cancer was obtained from the TIMER (http://timer.cistrome.org/) database [31], after which the GEPIA (Gene Expression Profiling Interactive Analysis; http://gepia2021.cancer-pku.cn/) [32] was used to perform correlation analysis, while UALCAN (http://ualcan.path.uab.edu) [33] online websites were used to evaluate the mRNA expression, gene expression correlation, and clinically relevant information of *ATAD2* in UCEC. In addition, we screened the *ATAD2* coexpressed genes using UALCAN. The Human Protein Atlas Database (HPA, https://www.proteinatlas.org/) [34] was used to evaluate the ATAD2 protein expression in endometrial cancer. TIMER and TISIDB (https://cis.hku.hk/TISIDB/index.php) [35] are two classic online sites for immunoassays with powerful immune infiltration analyses, which we applied for the evaluation of immune lymphocyte infiltration. Sangerbox (http://sangerbox.com/Tool) is a powerful website for raw letter analysis [36]; this tool used its GO and KEGG analysis functions. The list of extended full names and abbreviations of tumors are provided in Supplementary File 1.

### 2.2. Cell Introduction and Culture

The human endometrial endothelial cells (HEEC) and the endometrial cancer cell lines (HEC-1A, HEC-1B, Ishikawa, and RL95-2) were purchased from Genechem Co., Ltd. (Shanghai, China). HUVEC cells were generously provided by Dr. Yuan Liu. Dulbecco's modified Eagle's medium (DMEM) was used to cultivate the HEEC cells. Moreover, HEC-1A cells were cultured in McCoy's 5A medium. HUVEC cells were also cultured in Roswell Park Memorial Institute 1640 (RPMI-1640) medium. Moreover, both HEC-1B cells and Ishikawa cells were maintained in the MEM medium. DMEM/F-12 in a 1 : 1 ratio was considered the best alternative to consistently maintain the RL95-2 cells. All mediums were supplemented with 10% fetal bovine serum and 1% penicillin-streptomycin solution to provide nutrients and avoid contamination. The entire process of cell cultivation was performed in a 5% CO_2_ incubator at 37°C.

### 2.3. Cell Transfection and Infection

The lentiviruses overexpressing *ATAD2*, knocking down *ATAD2*, and their controls were packaged and produced by GENECHEM Shanghai, as per the manufacturer's instructions. Briefly, we first harvested HUVEC cells, seeded them into a 6-well plate (1 × 10^5^/well), and incubated the same under a 5% CO_2_ atmosphere at 37°C until reaching a confluence rate of 50%. Next, an appropriate concentration of lentivirus was injected into the wells and incubated for 12 h. Then, 1 mL of fresh complete-growth medium was added to the wells. After 48 h, puromycin was applied to screen the cells that were successfully transfected. The transfected rate was verified by western blotting.

### 2.4. Preparation of the Conditioned Medium

Ishikawa cells and HEC-1A cells in their respective logarithmic growth phase were subjected to continuous cell culture. When the confluence rate of 80% was achieved, the old complete medium was replaced with a fresh one and cultivated for 48 h. The liquid supernatant was then harvested and centrifuged for 10 min. Subsequently, a 0.22 *μ*m filter was used to clarify the liquid. The liquid was then stored at -20°C. The conditioned medium was prepared from 1640 complete medium and supernatant in a ratio of 2 : 1.

### 2.5. Wound Healing Assays

First, the HUVEC cell suspension was added to a 6-well plate and placed in an incubator; when the cells grew into a fused monolayer, the tips of a 200 *μ*L (4 × 10^5^/well) sterile pipettes were used to scrape them into the noncellular interstices. The wells were subsequently rinsed thrice with PBS, and different conditioned media were added to different wells. The wells were subsequently photographed by inverted microscopy at 0, 12, and 24 h of culture.

### 2.6. Western Blotting Analysis

The proteins were extracted by ice cracking. SDS-PAGE was used to separate the proteins. Subsequently, the target proteins were transferred onto a polyvinylidene difluoride membrane at low temperatures, followed by treatment with 5% milk for 50 min. Immediately afterward, the membranes were incubated overnight at 4°C with a primary antibody (*ATAD2*, 1 : 1000, #78568, Cell Signaling; *VEGFA*, 1 : 10000, ab52917, Abcam; and *GAPDH*, 1 : 5000, abs132004, Absin). In the following day, the membranes were removed and subjected to the process of PBST washing, treatment with a secondary antibody (anti-rabbit IgG, HRP-linked antibody, 1 : 5000, #7074 Cell Signaling), PBST rinsing, and, finally, exposure to the chromogenic apparatus by dropwise addition of the chromogenic solution for color development.

### 2.7. MTT Assay

Colorimetric 3-(4, 5-dimethylthiazol-2-yl)-2, 5-diphenyl-tetrazolium bromide (MTT) assay was performed to determine the extent of cell proliferation. First, in the logarithmic growth phase, HUVEC cells were inoculated into a 96-well plate (2 × 10^3^/well) for 24 h, followed by the addition of the conditioned medium and incubation at 37°C under a 5% CO_2_ incubator for 3 days. The cell viability was measured at 0, 24, 48, and 72 h after the addition of the conditioned medium. The prepared MTT reagent was added to a 96-well plate and incubated in the incubator for 4 h. Then, the medium was aspirated out and treated with 150 *μ*L of dimethyl sulfoxide (DMSO) to dissolve the blue-violet crystals. Finally, the 96-well plate was placed under the enzyme standard to read the absorbance at 490 nm.

### 2.8. Clone Formation Assays

HUVEC cells were cultured in a 6-well plate at the density of 600/well overnight, which made the cells adherent. In the next day, the complete medium was replaced with a conditional medium and incubated for 7–10 days. After colony formation, the colonies were fixed, washed, and stained to investigate clone formation.

### 2.9. Cell Migration Experiment

The transwell assay was performed to determine the migration capability of HUVEC in different conditioned media. The upper chamber of the apparatus was filled with 200 *μ*L of serum-free cell suspension containing 2 × 10^4^ HUVEC cells. Meanwhile, the lower chamber was added with 600 *μ*L of the conditioned medium and incubated at 37°C in a 5% CO2 for 24 h. Next, the chambers were removed and placed in 4% paraformaldehyde for 20–45 min for cell fixation. Subsequently, it was gently wiped with a cotton swab, and the excess cells in the upper chamber were rubbed with a cotton swab, washed twice with PBS, and stained with crystal violet for 15 min. The cells were finally observed under an inverted microscope, and pictures were randomly intercepted.

### 2.10. Cell Invasion Experiment

To investigate whether ATAD2 affected vascular endothelial cell invasiveness, we performed an invasion assay. The night before the experiment, the Matrigel was moved from a -20°C refrigerator into a 4°C refrigerator. On the second day, 70 *μ*L of the Matrigel solution prepared at the ratio of 1 : 8 was first added to each chamber and then the entire process was conducted on an ice plate. Next, the chambers were placed in a 24-well plate and incubated at 37°C for 30 min while the cell suspension was prepared. HUVEC cells were then resuspended in a serum-free 1640 medium, followed by counting to ensure that 120 *μ*L of the cell suspension containing 4 × 10^4^ cells was added to the upper layer of each chamber. In the lower chamber, 600 *μ*L of the conditioned medium was added. After 48 h, the chambers were removed and washed twice with PBS, and the unmigrated cells were removed from the surface of the membrane with cotton swabs, fixed in paraformaldehyde, and stained with crystal violet, followed by observation under a microscope and random photography for 5 fields of view.

### 2.11. Immunohistochemistry

Immunohistochemical staining was performed according to the standard protocol as described previously [16]. Briefly, the tissue sections were cut and baked in a 65°C oven for 2 h, followed by dewaxing and hydration in xylene and a gradient series of alcohol. After blocking in hydrogen peroxide, antigen repair was performed in an autoclave, washed thoroughly, and placed in a wet box with a drop of a primary antibody (ATAD2,1 : 400, bs-9110R, Bioss; VEGF, 1 : 50, 19003-1-AP, Proteintech; and CD31, 1 : 800, 66065-1-lg, Proteintech) in a 4°C refrigerator overnight. In the next day, the wet box containing the sections was rewarmed to room temperature for 20 min, incubated dropwise with horseradish peroxidase-labeled secondary antibody (ATAD2, PV-6001, ZSGB-BIO; VEGF, PV-6001, ZSGB-BIO; and CD31, PV-6002, ZSGB-BIO) for 30 min at the room temperature, diaminobenzidine (DAB) color development, hematoxylin staining, followed by dehydration and transparency, and then, subsequently, sealing and observation under a microscope.

Immunohistochemistry was scored as follows: brown or dark brown = 3, brown = 2, light brown or yellow = 1, and yellow or not visible = 0. The staining area was scored as 3 points for 100–76%, 2 points for 75–51%, 1 point for 50–26%, and 0 points for ≤25%. The product of the staining intensity score and the staining area score was considered the final score. A score ≥ 4 was assessed as positive, and <4 was assessed as negative. The MVD counting method was referred to using Weiner's [37] counting method, wherein the slice was observed under a low magnification field of view, and the area with a concentrated expression was selected as the hot spot, followed by observation under high magnification to count the number of microvessels in 5 different fields of view in a hot spot area; the average was considered as the final number of microvessels.

### 2.12. *In Vivo* Assay

The female BALB/C mice used in the experiments were purchased from Beijing Viton Lever Laboratory Animal Technology Co. and reared under suitable conditions, as directed. These mice were assigned to 4 groups: HEC-1A-NC (HEC-1A cells transferred into a blank plasmid, *n* = 3), HEC-1A-SH3 (HEC-1A cells with knockdown ATAD2, *n* = 3), Ishikawa-NC (Ishikawa cells transferred into a blank plasmid, *n* = 4), and Ishikawa-OE (ATAD2 overexpressing Ishikawa cells, *n* = 4); the cell suspension was prepared and 120 *μ*L (1.5 × 10^6^) cells were subcutaneously injected into each full-grown mouse (age: 6–8 weeks). After the formation of visible tumors, the mouse transplanted tumors were measured every 5 days. After 25 days, the mice were appropriately dissected to remove the tumor tissues for photography and subsequent experiments. The tumor volume was calculated using the following formula: length × width^2^ × 0.5. The mice were finally euthanized. All animal experiments are conducted with reference to the ARRIVE guidelines and based on the latest Chinese regulations and standards for the use of laboratory animals.

### 2.13. Statistical Analyses

All independent trials were repeated thrice, and the results were analyzed using IBM SPSS Statistics 26 software and GraphPad Prism software. Independent samples *t*-test was performed to compare the differences between the control and experimental groups two by two, with *P* < 0.05 (^∗^) indicating a significant difference and *P* < 0.01 (^∗∗^) and *P* < 0.005 (^∗∗∗^) indicating a highly significant difference.

## 3. Results

### 3.1. *ATAD2* Plays an Oncogenic Role in Endometrial Cancer

**Figure 1:**
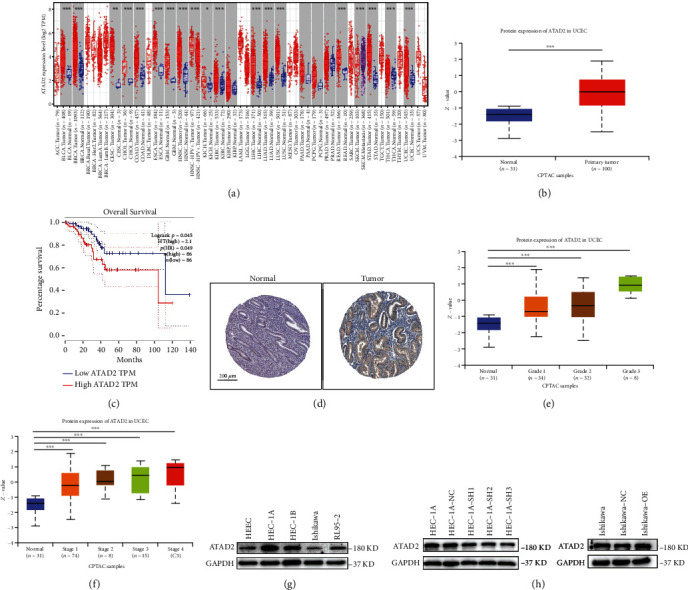
ATAD2 is highly expressed in endometrial cancer and correlates with the OS of the patient. (a) TIMER database assessment of *ATAD2* expression in pan-cancer. (b) UALCAN-based analysis of *ATAD2* mRNA expression in UCEC. (c) Impact of ATAD2 expression in UCEC on patient OS. (d) HPA analysis of ATAD2 protein expression in UCEC. (e, f) UALCAN assessment of ATAD2 and uterine endometrial cancer patients with clinical information correlation. (g) Expression of ATAD2 in human endometrial epithelial cells and four endometrial cancer cell lines. (h) Expression of ATAD2 in endothelial cancer cell lines after overexpression and knockdown of *ATAD2*.

Initially, the expression of *ATAD2* in pan-cancer was explored using the TIMER database, and the results are depicted in Figure 1(a). *ATAD2* was overexpressed in many tumors, including UCEC. Subsequently, the high expression of ATAD2 in UCEC was reverified using the UALCAN online sites (Figure 1(b)). The expression of the ATAD2 protein in patient tissues was downloaded from the HPA database, which indicated that ATAD2 was highly expressed in endometrial cancer tissues (Figure 1(d)). Furthermore, the correlation between ATAD2 and overall survival (OS) was evaluated in patients with UCEC using GEPIA results, which showed that ATAD2 overexpression decreased OS in patients (Figure 1(c)). ATAD2 expression was also correlated with the grade stage and stage classification of the patients (Figures 1(e) and 1(f)). All these results suggest that ATAD2 is an oncogene in endometrial cancer.

Later, the expression of ATAD2 in human endometrial cancer cell lines as well as normal human endometrial epithelial cells was determined using protein immunoblotting. The result showed that the expression of ATAD2 was significantly higher in endometrial cancer cell lines than in normal human endometrial cells, as illustrated in Figure 1(g). Moreover, Ishikawa, with the lowest ATAD2 expression, was selected for overexpression and HEC-1A, with the highest expression, for a knockdown. Western blot was used to verify the transfection rate. As shown in Figure 1(h), HEC-1A-SH3, with the best knockdown affection, was selected for subsequent experiments.

### 3.2. ATAD2 Promotes VEGF Expression in Endometrial Cancer

**Figure 2:**
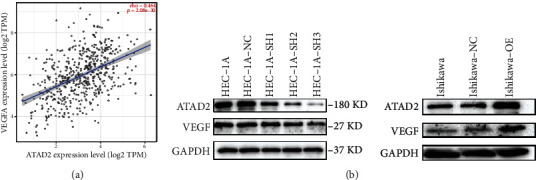
*ATAD2* promotes VEGF expression. (a) TIMER assessment of *ATAD2* and *VEGF* correlation in endometrial cancer. (b) VEGF expression in endometrial cancer cells overexpressing and knocking down *ATAD2*.

In the absence of blood vessels to supply oxygen, growth factors, and other nutrients, it is difficult for tumors to proliferate and progress. In particular, *VEGF* plays an important role in tumor neovascularization. Thus, the correlation between *ATAD2* and *VEGF* was evaluated using the TIMER website, and the results revealed a positive correlation between the two, as shown in Figure 2(a). Moreover, the expression of VEGF in endometrial cancer cell lines transfected with *ATAD2* was assessed, and the results are portrayed in Figure 2(b). It could be seen that VEGF expression was enhanced in endometrial carcinoma cells that overexpressed *ATAD2*. Also, VEGF expression was decreased in endometrial carcinoma cells in which *ATAD2* was knocked down. We, therefore, conclude that the expression of ATAD2 in endometrial cancer cells promotes the expression of VEGF.

### 3.3. ATAD2 Promotes the Proliferative Capacity of HUVEC

**Figure 3:**
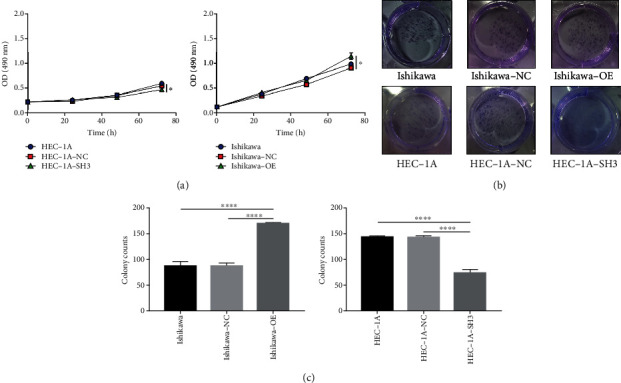
Supernatant liquid of transfected endometrial cancer cell lines affects the proliferative capacity of HUVEC. (a) MTT experiment: effect of the proliferation of HUVEC in culture with endometrial cancer supernatant transfected by *ATAD2*. (b) The cloning experiment reflects the proliferation between different groups. (c) Quantitative plots of clone formation in different groups.

To further explore the effect of *ATAD2* on endometrial tumor angiogenesis and cancer progression, supernatants of transfected Ishikawa, HEC-1A, and their controls as a conditioned culture were collected for culturing HUVEC. MTT and clone formation assays were used to detect the proliferation of HUVEC. As seen in Figure 3(a), the cell viability of HUVEC cultured under Ishikawa-OE-CM was significantly higher than that of the control compared with Ishikawa-CM or Ishikawa-NC-CM. On the contrary, the viability of cells cultured under HEC-1A-SH3-CM was inferior to that of its control. Furthermore, these results were verified using clone formation experiments in which HUVEC showed the greatest clone-forming ability in Ishikawa-OE-CM. Conversely, HUVEC in the HEC-1A group cultured under knockdown *ATAD2* supernatant (HEC-1A-SH3) conditions formed the fewest number of colonies (Figures 3(b) and 3(c)).

### 3.4. ATAD2 Affects the Migration and Invasion Abilities of HUVEC

**Figure 4:**
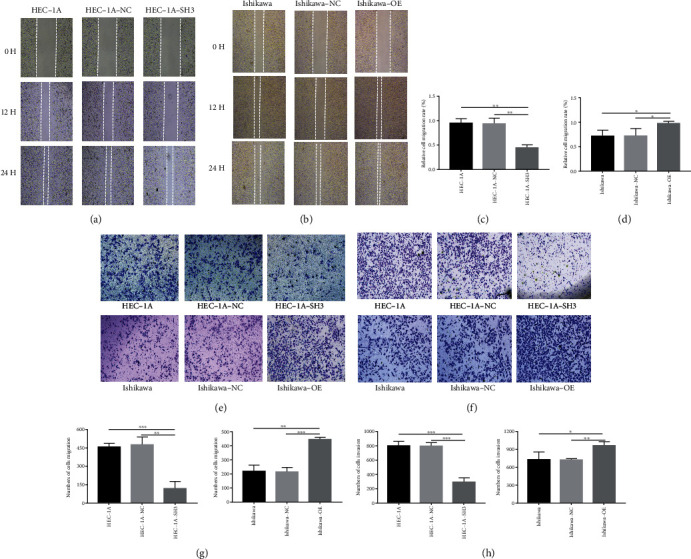
*ATAD2* affects the migration and invasion ability of HUVEC. (a) Effect of HEC-1A supernatant as conditioned medium on the migration ability of HUVEC (magnification, 40x). (b) Effect of supernatant of Ishikawa group on the migration ability of HUVEC (magnification, 40x). (c) Quantitative graph of the HEC-1A group scratch experiment. (d) Quantification of scratch experiments in Ishikawa group. (e) Differences in the migration ability of HUVEC cultured under different supernatant conditions (magnification, 100x). (f) Differences in the invasive ability of HUVEC under different groups of conditioned culture (magnification, 100x). (g) Quantitative graph of migration experiments. (h) Quantitative graph of the invasion experiment.

To further explore the role of *ATAD2* in tumor growth, transwell migration and wound healing assays were employed to assess the migration ability of HUVEC. The results of the wound healing assay indicated that the wound healing efficiency of HUVEC cultivated under Ishikawa-OE-CM was the highest (Figures 4(b) and 4(d)). On the contrary, the wound healing capability of HUVEC cultivated under HEC-1A-SH3-CM was greatly suppressed (Figures 4(a) and 4(c)). Moreover, the transwell migration assay confirmed this result. More HUVEC migrated toward Ishikawa-OE-CM compared with Ishikawa-CM and Ishikawa-NC-CM at 24 h. In addition, the migration ability of HUVEC cultivated under HEC-1A-SH3-CM was markedly retarded (Figures 4(e) and 4(g)). Collectively, it could be inferred that *ATAD2* exerted some influence on the migration ability of HUVEC. Subsequently, the role of *ATAD2* on the aggressive ability of HUVEC was examined using the transwell assay. As shown in Figures 4(f) and 4(h), HUVEC cultured in Ishikawa-OE conditioned medium was different from those cultured in Ishikawa or Ishikawa-NC conditioned medium. The invasive ability was the highest in Ishikawa-OE-CM. Nevertheless, the invasive ability of the HEC-1A-SH3-CM group was less than that of HEC-1A-NC-CM and HEC-1A-CM.

### 3.5. Upregulation of *ATAD2* Promotes Tumor Growth and Angiogenesis

**Figure 5:**
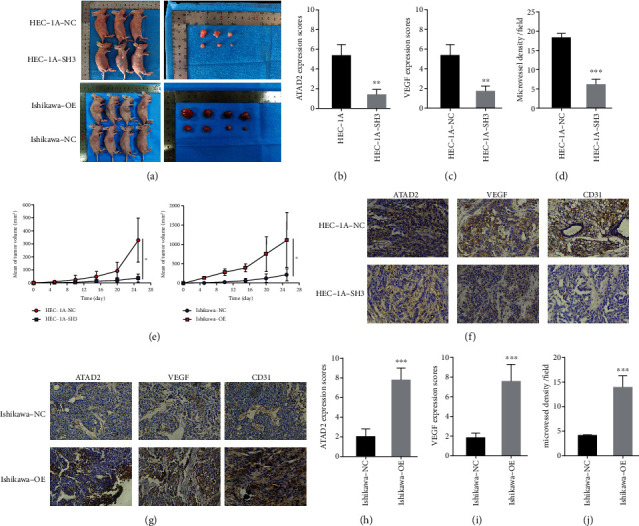
Upregulation of *ATAD2* promotes tumor growth and angiogenesis. (a) Transplant tumor growth in mice after injection of *ATAD2* overexpression, knockdown, and control of endometrial cancer cells. (b–d) Expression of ATAD2, VEGF, and MVD in knockdown *ATAD2* tumors. (e) Mouse transplant tumor growth curve. (f) Immunohistochemical images corresponding to knockdown of *ATAD2* (magnification, 200x). (g) The picture of immunohistochemistry of transplanted tumor when *ATAD2* was overexpressed above (magnification, 200x). (h–j) Overexpression of *ATAD2* increases the expression of VEGF and MVD in transplanted tumor models.

To further investigate the influence of *ATAD2* on oncogenesis, endometrial carcinoma cells with normal, insufficient, and upregulated *ATAD2* expression were inoculated into the skin of nude mice and a mouse transplantation tumor model was constructed. The tumor size was measured periodically, and the mice were euthanized after 25 days to dissect the tumor tissue. The tumors in the Ishikawa-OE group overexpressing *ATAD2* were found to grow significantly faster than those in the control group. In contrast, the tumors in mice injected with *ATAD2*-deficient cells (HEC-1A-SH3) were significantly smaller and grew much slower than those in the HEC-1A control group (Figures 5(a) and 5(e)). Immunohistochemical experiments were subsequently performed on the embedded wax blocks of transplanted tumors to further confirm the relationship between *ATAD2* and tumor angiogenesis at the tissue level. The results demonstrated that both VEGF expression and microvessel density were reduced in transplanted tumors after the knockdown of *ATAD2* (Figures 5(b)–5(d) and 5(f)). Conversely, with the increase in ATAD2 expression, VEGF as well as microvessel density showed high expression status (Figures 5(g)–5(j)). In summary, *ATAD2* promotes angiogenesis and tumor cell growth in endometrial cancer.

### 3.6. *ATAD2* Is Correlated with Tumor Immune Infiltration

**Figure 6:**
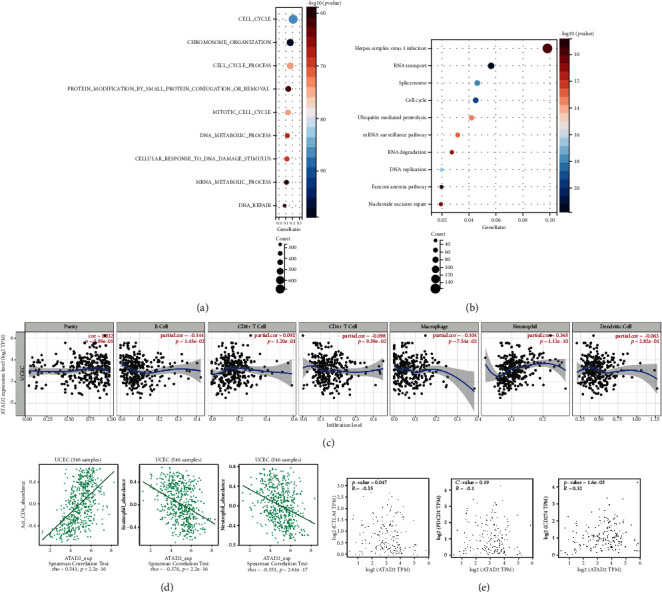
*ATAD2* is associated with TIL infiltration in UCEC. (a) GO enrichment analysis of the biological processes of *ATAD2* coexpressed genes. (b) KEGG enrichment analysis of the signaling pathways of *ATAD2* coexpressed genes. (c) TIMER online website assessing *ATAD2* correlation with immune infiltration in UCEC. (d) Three lymphocytes most significantly correlated with *ATAD2* immune infiltration analysis in UCEC based on TISIDB. (e) GEPIA analysis of *ATAD2* correlation with classical immune checkpoint inhibitors.

To gain more insights into the role of *ATAD2* in endometrial cancer, 3794 coexpressed genes with an *ATAD2* correlation coefficient of ≥0.3 were searched in UALCAN. Later, KEGG and GO analyses were performed using Sangerbox. The results are shown in Figure 6(a). GO analysis revealed that the biological processes mainly involved were the cell cycle, chromosome organization, and cell cycle process. In the KEGG enrichment analysis, the top 10 were selected for visualization (Figure 6(b)). The genes were mainly enriched in herpes simplex virus-1 (HSV-1) infection, RNA transport, spliceosome cell cycle, and other pathways, with the HSV-1 infection pathway being the major one. The relationship between *ATAD2* and lymphocyte infiltration in UCEC was then evaluated using the TIMER online database. The results are shown in Figure 6(c). A positive correlation was observed between *ATAD2* and tumor purity (cor = 0.032, *P* = 5.89e − 01). In addition, ATAD2 expression levels were positively correlated with neutrophils (cor = 0.365, *P* = 1.12*e* − 10) and CD8+ T cells (cor = 0.092, *P* = 1.20*e* − 01). On the contrary, B cells (cor = −0.144, *P* = 1.45*e* − 02), CD4+ T cells (cor = −0.098, *P* = 9.59*e* − 02), macrophages (cor = −0.104, *P* = 7.54*e* − 02), and dendritic cells (cor = -0.063, *P* =2.82*e*-01) were significantly negatively correlated. The correlation between *ATAD2* and lymphocyte infiltration was subsequently assessed using TISIDB, and the three most relevant lymphocytes, i.e., Act CD4+ T cells, neutrophils, and eosinophils, are screened (Figure 6(d)).

Immunotherapy based on immune checkpoint inhibitors plays a crucial role in the treatment of tumors. Currently used immunotherapies mostly target T-cell immunomodulators. Hence, the correlation between *ATAD2* and these three immune checkpoints was analyzed in UCEC using GEPIA. The results showed that *ATAD2* had a significant positive correlation with *programmed death receptor-1 ligand* (*PDL-1*), a negative correlation with *cytotoxic T-lymphocyte associated protein 4* (*CTLA4*), and no correlation with *programmed cell death 1* (*PD1*) (Figure 6(e)). These results imply that *ATAD2* is associated with immune infiltration in UCEC.

## 4. Discussion

With technological advancements, histology is playing an increasingly important role in cancer, which can give oncologists a complete picture of cancer dynamics [38]. However, with the advent of precision medicine, oncologists have gradually shifted their focus on the disease from the population to the individual level. This is a huge step forward in healthcare. In 1995, when Calman-Hine proposed improving the clinical outcomes of cancer care in the UK, the UK Department of Health introduced the multidisciplinary team (MDT) model for the management of chronic diseases, which has greatly improved patient survival [39]. This model has been used in recent years in gynecologic oncology to enhance patient survival via collaboration among gynecologists, oncologists, geneticists, and molecular biologists [40]. In ovarian cancer, especially in advanced stages, the complexity and severity of the disease necessitate that pathologists, imaging experts, and oncologists share clinical information and develop optimal treatment plans for the management of the disease [41–43]. Therefore we should focus on the value of histological science and a multidisciplinary approach to the treatment of gynecologic malignancies. However, a definitive diagnosis of the disease is the foremost requirement. Currently, the incidence of endometrial cancer is increasing, but there is a lack of effective early diagnostic indicators [44]. Hence, this study is mainly intended to explore new diagnostic indicators and therapeutic targets for endometrial cancer.

In this work, supernatants of overexpressed and knocked down *ATAD2* and their controls as conditioned media were used to culture HUVEC. The results of MTT and clone formation assays showed that the conditioned media overexpressing *ATAD2* promoted the proliferation of HUVEC more than their controls. Moreover, the conditioned medium of endometrial cancer cells with knocked down *ATAD2* inhibited the proliferation of HUVEC to a greater extent than its control. This result is indeed encouraging. Subsequently, the effects of transfected cell supernatant on the invasive ability of HUVEC were investigated using scratch, migration, and invasion assays. The results demonstrated that the overexpression of *ATAD2* in the endometrial cancer cell supernatant significantly enhanced the invasive ability of HUVEC. Most importantly, the western blot technique verified the positive correlation between ATAD2 and VEGF expressions in endometrial cancer. Furthermore, *in vivo* animal experiments established that *ATAD2* promoted tumor growth. Also, the elevated expression of ATAD2 led to the high expression of VEGF and CD31, which represents microangiogenesis. Thus, *ATAD2* can influence the expression of VEGF and promote intratumor angiogenesis.

To further explore the potential role of ATAD2 in endometrial cancer, 3,794 ATAD2 coexpressed genes were searched in UALCAN, followed by GO and KEGG analyses using Sangerbox. The findings revealed that the biological functions of these genes were mainly related to cell cycle and chromosome organization. KEGG enrichment analysis indicated that these genes were chiefly involved in the HSV-1 pathway. It has been shown that the HSV-1 infection pathway mainly affects the immune system, for example, by influencing the clonal proliferation of CD4+ T cells and triggering immune escape [45]. This observation suggests that the molecular mechanism of *ATAD2* may be related to immune inflammation-related functions. However, immunotherapy for gynecologic tumors has advanced dramatically in recent years, especially for advanced and refractory endometrial cancer. A recent clinical trial showed that immunotherapy combined with antiangiogenic therapy greatly prolongs OS and disease-free survival in patients with advanced endometrial cancer [46]. In this study, the relationship between *ATAD2* and immune lymphocyte infiltration was evaluated using the bioinformatics database, which showed that *ATAD2* is not only associated with angiogenesis but also with tumor immune infiltration. Thus, *ATAD2* is likely to serve as a promising therapeutic target.

Our study has certain limitations. The correlation between *ATAD2* and immunity is based only on public database studies using bioinformatics tools, and there is a lack of basic experiments to verify the mechanism of correlation. Therefore, we intend to continue our follow-up study with a detailed experimental design to validate the predicted results. In a nutshell, the findings of this study are useful for the early screening of endometrial cancer and provide a new direction to effectively control the malignant behavior of endometrial cancer.

## 5. Conclusion

In summary, it is an encouraging result that *ATAD2* promotes *VEGF* expression as well as tumor growth and angiogenesis in endometrial cancer. More interestingly, *ATAD2* is also associated with immune infiltration in UCEC. Thus, *ATAD2* has the potential to be used as a molecular diagnostic marker and potential therapeutic target in the diagnosis and treatment of endometrial cancer and as an early warning sign of malignancy. However, there are some limitations in this study. First, we only explored the correlation between *ATAD2* and immunity in bioinformatics-based public database studies without experimental validation. Second, although we constructed overexpression and knockdown cell lines to test our hypothesis, we only selected a single cell line with high/low *ATAD2* expression for validation. Hence, in the future, we will conduct more in-depth use of basic research to explore the deeper mechanisms by which *ATAD2* affects endometrial cancer angiogenesis and immune infiltration.

## Data Availability

The datasets used and analyzed during the current study are available from the corresponding author on reasonable request.

## Supplementary Materials

Supplementary Materials The extended full names and abbreviations of the various tumors in Figure 1(a) are described in detail in Supplementary File 1.
