# Supplementary material for: ATAD2 Upregulation Promotes Tumor Growth and Angiogenesis in Endometrial Cancer and Is Associated with Its Immune Infiltration
Source: Dis Markers. 2022 Nov 28;2022:2334338. doi: 10.1155/2022/2334338 (PMC9722300; doi:10.1155/2022/2334338)
Supplement: Supplementary Materials — The extended full names and abbreviations of the various tumors in Figure 1(a) are described in detail in Supplementary File 1. [file 2334338.f1.pdf]

Abbreviation	Full Name
ACC	Adrenocortical Carcinoma
BLCA	Bladder Urothelial Carcinoma
BRCA	Breast Invasive Carcinoma
CESC	Cervical and Endocervical Cancer
CHOL	Cholangiocarcinoma
COAD	Colon Adenocarcinoma
DLBC	Diffuse Large B-cell Lymphoma
ESCA	Esophageal Carcinoma
GBM	Glioblastoma Multiforme
HNSC	Head and Neck Cancer
KICH	Kidney Chromophobe
KIRC	Kidney Renal Clear Cell Carcinoma
KIRP	Kidney Renal Papillary Cell Carcinoma
LAML	Acute Myeloid Leukemia
LGG	Lower Grade Glioma
LIHC	Liver Hepatocellular Carcinoma
LUAD	Lung Adenocarcinoma
LUSC	Lung Squamous Cell Carcinoma
MESO	Mesothelioma
OV	Ovarian Serous Cystadencarcinoma
PAAD	Pancreatic Adenocarcinoma
PCPG	Pheochromocytoma and Paraganglioma
PRAD	Prostate Adenocarcinoma
READ	Rectum Adenocarcinoma
SARC	Sarcoma
SKCM	Skin Cutaneous Melanoma
STAD	Stomach Adenocarcinoma
TGCT	Testicular Germ Cell Tumors
THCA	Thyroid Carcinoma
THYM	Thymoma
UCEC	Uterine Corpus Endometrial Carcinoma
UCS	Uterine Carsinosarcoma
UVM	Uveal melanomas

Note: This table is a list of full names and abbreviations of tumor extensions
